# Hematopoietic cytokines as therapeutic players in early stages Parkinson’s disease

**DOI:** 10.3389/fnagi.2015.00126

**Published:** 2015-07-03

**Authors:** Kyle Farmer, Christopher Rudyk, Natalie A. Prowse, Shawn Hayley

**Affiliations:** Department of Neuroscience, Carleton UniversityOttawa, ON, Canada

**Keywords:** Parkinson’s disease, early stage, cytokines, granulocyte macrophage-colony stimulating factor, erythropoietin

## Abstract

Parkinson’s disease (PD) is a devastating age related neurodegenerative disease that is believed to have a lengthy prodromal state. It is critical to find methods to harness compensatory recovery processes in order to slow or prevent the eventual progression of clinical symptoms. The current perspective paper argues that immune system signaling molecules represent such a promising therapeutic approach. Two cytokines of interest are granulocyte macrophage-colony stimulating factor (GM-CSF) and erythropoietin (EPO). These hematopoietic cytokines have been protective in models of stroke, neuronal injury, and more recently PD. It is our belief that these trophic cytokines can be used not only for cell protection but also regeneration. However, success is likely dependent on early intervention. This paper will outline our perspective on the development of novel trophic recovery treatments for PD. In particular, we present new data from our lab suggesting that EPO and GM-CSF can foster neural re-innervation in a “mild” or partial lesion PD model that could be envisioned as reflecting the early stages of the disease.

## Parkinson’s Disease: Animal Model of Early Stages

Parkinson’s disease (PD) is characterized by a loss of dopamine (DA) neurons within the nigrostriatal pathway and the presence of Lewy body pathological protein aggregates (Farrer et al., 2001; Sherer et al., 2001). Clinically, PD is diagnosed based on tremors within distal limbs, muscle rigidity, and bradykinesia. By the time patients present with these motor symptoms, there has already been significant degeneration of DA neurons, with up to an 80% loss of striatal DA innervation (Bezard et al., 2001). There are also extensive non-motor symptoms, which present long before the cardinal motor symptoms (McDonald et al., 2003).

Current PD treatments only manage symptom severity and are not able to reverse or even appreciably slow the neurodegenerative processes. Thus, it is of interest to investigate potential treatments that could stabilize these surviving neurons and possibly induce some degree of neuronal recovery. It might be advantageous to target processes linked to the early or prodromal stages of PD, as neuronal plasticity would likely be more amenable to modulation at such times. However, models of early stage PD are less common and not as well understood as the late stage typically used. The neurotoxin, 6-hydroxydopamine (6-OHDA), is routinely used to induce PD-like pathology, inducing a loss of substantia nigra pars compacta (SNc) DA neurons and downstream striatal terminals (Alvarez-Fischer et al., 2008). 6-OHDA infused directly into the SNc rapidly produces a robust degeneration of SNc DA neurons, coupled with striatal DA depletion within 48–72 h (Blandini et al., 2008; Thiele et al., 2012). However, this method has the obvious caveat of not reflecting the chronic slow course of degeneration. A more progressive lesion has been observed with lower doses of 6-OHDA infused into the striatum rather than SNc. Indeed, intra-striatal 6-OHDA administration induced a lesion, which gradually increased in size over several weeks (Sauer and Oertel, 1994) and more closely mimicked the progression from early to later stages of PD.

## Novel Treatment Strategies

One exciting new avenue for treating PD involves the use of trophic factors to stabilize neuronal viability and even promote some degree of recovery. In fact, recent studies have revealed a reduction of brain derived neurotrophic factor (BDNF) within the SNc of PD patients (Mogi et al., 1999; Salehi and Mashayekhi, 2009). Accordingly, BDNF can promote the survival and differentiation of mesencephalic DA neurons, as well as protect against the DA toxicants, 1-methyl-4-phenyl-1,2,3,6-tetrahydropyridine (MPTP) and 6-OHDA (Murer et al., 2001). Likewise, glial derived neurotrophic factor (GDNF), has also emerged as a potential candidate for neuroprotection in PD patients, based on success in various animal models (Fox et al., 2001; Ai et al., 2003). However, the improvements observed in clinical trials were restricted to the immediate area surrounding the site of infusion (Gill et al., 2003) and a randomized placebo-controlled study was unsuccessful at replicating these beneficial effects (Lang et al., 2006). Moreover, BDNF and GDNF do not readily cross the blood brain barrier (BBB) and have numerous side effects (Pezet and McMahon, 2006; Pilakka-Kanthikeel et al., 2013).

Finding well-tolerated factors with trophic properties, which cross the BBB represents a considerable challenge. Two cytokines that may hold potential therapeutic significance are the hematopoietic cytokines, erythropoietin (EPO) and granulocyte macrophage-colony stimulating factor (GM-CSF). Indeed, GM-CSF had protective effects in models of Alzheimer’s disease (Boyd et al., 2010), and in MPTP and paraquat models of PD (Kim et al., 2009; Mangano et al., 2011). Moreover, GM-CSF administration induced spontaneous axonal regeneration and functional recovery from traumatic spinal cord injury (Ha et al., 2005; Bouhy et al., 2006) and reduced infarct volume following ischemia (Nakagawa et al., 2006; Schäbitz et al., 2008). Similarly, EPO has been investigated extensively for use in stroke, traumatic head injury and more recently, in toxin based animal models of PD (Sargin et al., 2010; Merelli et al., 2013; Bond and Rex, 2014). EPO was also shown to protect hippocampal neurons from stressor-induced apoptosis, and increased adult hippocampal neurogenesis (Merelli et al., 2015).

GM-CSF and EPO have well-documented trophic actions in the periphery and can infiltrate and accumulate within the brain (Enzler and Dranoff, 2003). Receptors for GM-CSF and EPO have been found on mature DA neurons and neural progenitor cells, suggesting that they might influence adult neuronal functioning, as well as stimulate maturation (Kim et al., 2004; Ha et al., 2005).

The peripheral function of GM-CSF is to promote the differentiation and maturation of innate immune cells, and it is routinely administered to cancer patients to modify neutrophil production (Dale et al., 1998). Similarly, EPO has potent mitogenic effects on immune cells, as well as red blood cells and is routinely prescribed for anemia and in the context of certain cancer treatments (Debeljak et al., 2014). Thus, both also have well established clinical track records.

## GM-CSF and EPO Promote Striatal Re-Innervation

It is thought that a slow progressive “wave” of neurodegeneration occurs over many years before a critical threshold of neuronal loss is reached and clinical PD pathology is manifested. Accordingly, it is of interest to study the effects of potential treatments while the disease is still in an early or possibly even a prodromal stage. In the present paper, it was of interest to assess whether EPO or GM-CSF treatment could influence striatal innervation following the establishment of a partial or “mild” lesion that might be analogous to the early onset of the disease. To this end, male Sprague Dawley rats received a single intra-striatum (1.0 mm anterior, 3.0 mm lateral, 5.0 mm ventral relative to bregma) infusion of 6-OHDA (20 μg). Animals were then randomly divided into three groups (*n* = 7): Saline, EPO, and GM-CSF. On post-6-OHDA infusion Days 13 and 28, the animals received an intraperitoneal injection of either saline, recombinant human EPO solution (rhEPO; 50 μg/kg) or recombinant rat granulocyte-macrophage colony-stimulating factor solution (rat GM-CSF; 10 μg/kg). Animals were sacrificed on Day 30.

**Figure 1:**
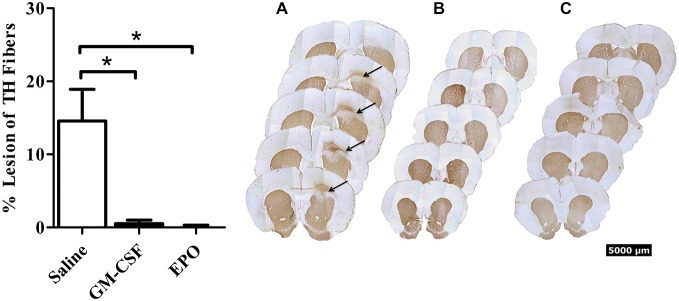
**Thirty days after the intra-striatal infusion of 6-OHDA, saline treated animals (graph—white bars; A) had a modest but statistically significant loss of TH+ striatal fibers.** The GM-CSF (graph—black bars; **B**) and EPO (graph—black bars; **C**) treated animals displayed no visible lesion at the 30-day sacrifice time. Data is expressed as mean ± 1 SEM, *p < 0.01.

**Figure 2:**
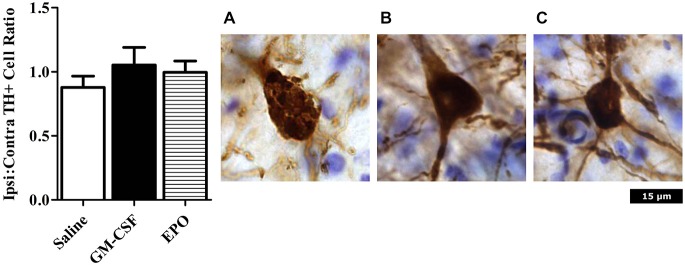
**Neurons were counted in an unbiased manner using MBF Stereo investigator optical fractionator probe.** There were no significant differences between treatment groups (left graph). Photomicrographs are representative of animals treated with **(A)** Saline; **(B)** GM-CSF; or **(C)** EPO in conjunction with 6-OHDA infusion. The saline (but not GM-CSF or EPO) treated rats that received 6-OHDA displayed TH+ neurons with an abnormal shaped nucleus, with a reduction of projections. Images were taken at 40 × magnification.

As shown in Figure 1, we did indeed find that a modest [~10% of striatal area, as determined using tyrosine hydroxylase (TH) staining] lesion was induced in rats following intra-striatal infusion of a single moderate dose of 6-OHDA. However, no significant neuronal loss was evident within the SNc using this relatively mild paradigm, although it is possible that some of these SNc neurons would eventually die if we explored longer time intervals. Indeed, many surviving neurons had irregular shaped soma (Figure 2).

Importantly, the two EPO and GM-CSF injections that were given *after* the presumed establishment of the lesion (i.e., on Days 13 and 28 following 6-OHDA) provoked a significant recovery (presumed re-innervation) of striatal terminals. The statistical analysis revealed a significant treatment effect, *F*_(2,15)_ = 10.7, *P* < 0.01, with regards to striatal lesion size (Figure 1), such that the cytokines prevented the striatal lesion by Day 30 following 6-OHDA.

The present findings are consistent with our own previous data and those of others showing beneficial effects of GM-CSF (Mangano et al., 2011; Kosloski et al., 2013) and EPO (Xue et al., 2007; Dhanushkodi et al., 2013; Qi et al., 2014) in toxicant animal models. However, since GM-CSF or EPO were administered after lesion establishment, the effects of the cytokine treatments in the current study would be expected to reflect some degree of recovery involving DA fiber re-growth rather than the prevention of fiber loss in the first place. This is a particularly novel finding given that the majority of studies typically focus on neuroprotective effects, rather than addressing the more clinically relevant issue of promoting recovery following some degree of neuronal damage.

## Impact and Future Directions

It is important to underscore that the 6-OHDA paradigm presently used provoked a very modest loss of striatal terminals and future studies are required to ascertain whether GM-CSF and EPO might also have reparative properties in PD models with more significantly sized lesions. Nonetheless, GM-CSF and EPO may be ideal trophic treatment candidates based on their biological profiles, preclinical data and track record of clinical applicability. Mechanistically, these cytokines are potent inducers of BDNF and GDNF (Bouhy et al., 2006; Mengozzi et al., 2012), which we posit to be fundamental for their beneficial neural consequences. Targeting trophic processes to boost plasticity may be a critically important shift in treatment modalities away from failed attempts to translate neuroprotective approaches to the clinic; this strategy would also work well in tandem with recent efforts to identify biomarkers of disease state.

## Author Contributions

CR and SH conceived the study. KF, CR, and SH designed the study. KF and CR performed the *in vivo* procedures. KF performed the immunohistochemical stains and data analysis. NP performed the stereological SNc counts. KF and SH prepared the manuscript. All authors have read and approved the final manuscript.

## Conflict of Interest Statement

The authors declare that the research was conducted in the absence of any commercial or financial relationships that could be construed as a potential conflict of interest.
